# Extreme heat reduces host and parasite performance in a butterfly–parasite interaction

**DOI:** 10.1098/rspb.2023.2305

**Published:** 2024-01-17

**Authors:** Isabella G. Ragonese, Maya R. Sarkar, Richard J. Hall, Sonia Altizer

**Affiliations:** ^1^ Odum School of Ecology, College of Veterinary Medicine, University of Georgia, Athens, GA 30602, USA; ^2^ Center for the Ecology of Infectious Diseases, College of Veterinary Medicine, University of Georgia, Athens, GA 30602, USA; ^3^ Department of Infectious Diseases, College of Veterinary Medicine, University of Georgia, Athens, GA 30602, USA; ^4^ College of Biological Sciences, University of Minnesota, St Paul, MN 5455, USA

**Keywords:** *Danaus plexippus*, *Ophryocystis elektroscirrha*, temperature, thermal ecology, infection, immune defence

## Abstract

Environmental temperature fundamentally shapes insect physiology, fitness and interactions with parasites. Differential climate warming effects on host versus parasite biology could exacerbate or inhibit parasite transmission, with far-reaching implications for pollination services, biocontrol and human health. Here, we experimentally test how controlled temperatures influence multiple components of host and parasite fitness in monarch butterflies (*Danaus plexippus*) and their protozoan parasites *Ophryocystis elektroscirrha*. Using five constant-temperature treatments spanning 18–34°C, we measured monarch development, survival, size, immune function and parasite infection status and intensity. Monarch size and survival declined sharply at the hottest temperature (34°C), as did infection probability, suggesting that extreme heat decreases both host and parasite performance. The lack of infection at 34°C was not due to greater host immunity or faster host development but could instead reflect the thermal limits of parasite invasion and within-host replication. In the context of ongoing climate change, temperature increases above current thermal maxima could reduce the fitness of both monarchs and their parasites, with lower infection rates potentially balancing negative impacts of extreme heat on future monarch abundance and distribution.

## 1. Introduction

Ongoing climate change is expected to have striking impacts on patterns of infectious disease in wildlife [1,2]. These effects can manifest when elevated temperatures influence external parasite transmission stages or the biology of arthropod vectors (e.g. [3]) and will also be important for parasites infecting ectothermic hosts such as insects, which themselves are temperature-sensitive [1,4]. A warmer world is predicted to include widespread heatwaves and a loss of nighttime cooling, exposing many organisms to extended periods of high temperatures [5–9]. Already, climate warming is associated with geographic range shifts, population declines and modified phenology in insects, impacting the services and disservices that they provide [4,10–12]. Given their importance as agricultural pests, biocontrol agents, and vectors of emerging pathogens, most studies of temperature effects on insect–parasite interactions to date have focused on insects of medical and economic concern [13–15]. At the same time, it is known that pathogens can cause reduced fitness and population declines across a broad range of insect species, including those with ecological importance as pollinators, herbivores, predators and decomposers [16,17]. Thus, there is a crucial need to understand the consequences of climate change for insect–pathogen population dynamics more broadly.

Past work shows that insect and parasite traits relevant to infection, such as immune defence, development rate and survival, can respond to temperature in different ways [4,14,18–22]. In some cases, heat stress experienced by hosts could exacerbate susceptibility to and impacts of infection; in other cases, hosts might benefit from reduced parasite replication at very high temperatures [23–26]. Insect innate immunity includes cellular defences (executed by haemocytes responsible for encapsulation, phagocytosis and secretion of molecules involved in lysis and melanization [27,28]) and humoral immunity, which relies on the circulation of immune peptides and phenoloxidases (PO), enzymes vital for melanin production [28,29]. In some insects, higher temperatures lead to increased haemocyte concentrations [30], elevated phenoloxidase, and stronger antibacterial responses [29]. By contrast, other insects demonstrate higher immune function at cooler temperatures [31,32]. Similarly, temperature can have differing effects on internal parasite stages. For some parasites, within-host replication is limited most by cool temperatures [19], whereas other parasites cannot tolerate heat. For example, fungal pathogen development in locusts is inhibited when the insect hosts thermoregulate, driving their body temperatures to ≥40°C in a behavioural fever [30]. Thus, to assess the potential impacts of changing global temperatures on insect diseases, researchers must understand how both host and parasite traits respond to temperature gradients and extremes.

Monarch butterflies (*Danaus plexippus*) and their protozoan parasite *Ophryocystis elektroscirrha* (hereafter, OE) are well-suited for studying how host and parasite responses to temperature can impact infection outcomes. North American monarchs perform an iconic annual migration east of the continental divide: adults overwinter in Mexico and recolonize their breeding range (as far north as southern Canada) in successive generations, throughout which they experience regional and seasonal temperature variation. Monarchs also populate subtropical and tropical regions of the globe [33–35], and OE infections have been observed in every population of monarchs examined to date [34,36,37]. Parasitism lowers monarch fitness through reduced larval survival, lower eclosion and mate-finding success, smaller adult size, shorter adult lifespans and lower flight performance [38–40].

Previous work demonstrates that temperature can impact immature and adult monarch survival [41,42], development rate [42,43] and reproduction [42,44], which are also traits that influence monarch–parasite interactions. Larval monarch survival shows a unimodal relationship with temperature, peaking around 28°C [41]. Monarchs develop more quickly as temperature rises, but exposure to extreme heat (>38°C) can lengthen development times [42,43]. Past research on OE infectious stages (oocysts or ‘spores’ that persist on the outside of hosts or other substrates) showed that the parasites remain viable across a range of temperatures (3–32°C) for up to two weeks, and that longer-term spore viability decreases markedly with increasing temperature [45]. Taken together, these findings suggest that hot temperatures could be harmful to both monarchs and their parasites. Notably, the Thermal Mismatch Hypothesis [2,23] predicts that as local conditions shift away from the host's optimal temperature, parasites will outperform hosts, increasing infection probability or intensity. This assumes that parasites have a wider thermal range than their hosts and that host immunity and other anti-parasite defences decline away from the optimum. For monarchs, a crucial need remains to investigate how temperature impacts within-host parasite development, and whether temperature-dependent host traits such as development and immunity affect infection outcomes.

**Figure 1.**
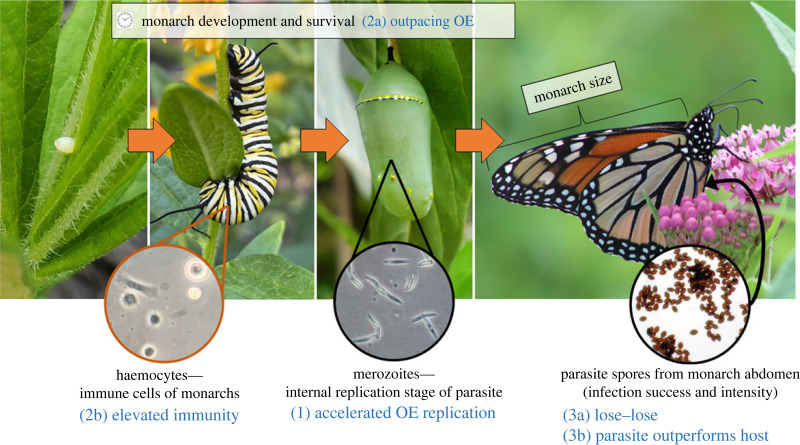
Possible temperature-dependence of within-host traits and infection outcomes as measured across the monarch life cycle. Numbers relate to predictions made in table 1. Data on monarch development time and immunity (including haemocyte concentration) were collected to address whether monarch traits could explain differences in infection across temperature. To assess *Ophryocystis elektroscirrha* (OE) replication, merozoite concentrations in pupae and OE spore loads on adults were measured. Infection success, parasite replication, host size and survival were tracked to determine whether any temperature treatments were detrimental to monarchs or OE.

Here we investigate how temperature impacts monarch infection outcomes, including infection probability, parasite load (a proxy for within-host replication) and fitness consequences of infection. We also examine associations between temperature and host innate immunity and development rate as possible mechanisms to explain infection outcomes. Thermal performance curves (TPCs), which describe the response of biological rates to temperature, are a useful framework for understanding how insects and their parasites respond to climate warming. Informed by the metabolic theory of ecology [46], many TPCs for ectotherms are nonlinear and unimodal, but individual traits (e.g. host immunity and parasite development rate) respond uniquely, with different thermal minima, maxima and optima [18,47–49]. We predicted that (1) below monarchs' thermal optimum for pre-adult survival (approx. 28°C; [41]), within-host replication of the parasite would increase with temperature. Alternatively, warmer temperatures approaching the host's optimum could lower spore load, owing to either (2a) increasing host development rate, which yields less time for parasite replication, or (2b) elevated host immunity. Above the host's thermal optimum, we predicted that either (3a) higher temperatures will lower metrics of host performance (e.g. survival, size), within-host parasite replication, and infection probability, or (3b), consistent the Thermal Mismatch Hypothesis, host immunity and performance metrics will decline more steeply with temperature than parasite replication, worsening infection outcomes for the host (figure 1 and table 1).

**Table 1.** Predictions of mechanisms driving temperature-dependent infection outcomes and the traits measured to address the predictions. OE, *Ophryocystis elektroscirrha*.

prediction	traits measured to assess impacts on infection outcomes
(1) accelerated OE replication: parasite within-host replication increases with temperature, yielding **higher** infection success and intensity	merozoite concentration in pupae (parasite replication)
(2a) outpacing parasites: host development speeds up with temperature, allowing monarchs to outpace OE and yielding **lower** infection success and intensity	monarch development time to pupation and adult emergence
(2b) elevated immunity: host immune defences increase with temperature, resulting in **lower** infection success and intensity	haemocyte concentration and phenoloxidase activity in larvae and pupae
(3a) lose–lose: at the hottest temperatures, performance of both OE and monarchs **decreases**	parasite replication, infection success, host size and survival
(3b) parasite outperforms: at the hottest temperatures, OE performance is higher relative to monarchs, leading to **higher** infection success and intensity	parasite replication, infection success, host size and survival

## 2. Material and methods

### (a) Biology of *Ophryocystis elektroscirrha* transmission

Parasite transmission occurs when dormant OE spores are ingested by monarch larvae feeding on contaminated eggs or milkweed (*Asclepias* spp.) host plants. After ingestion, spores lyse, penetrate the gut wall and undergo several stages of asexual reproduction [50]. During the host pupal stage, parasite spores form among the scales of the developing butterfly. When adults emerge, they are covered with millions of dormant spores, particularly on the abdomen [51]. These spores are then deposited onto eggs and milkweed during oviposition or nectaring and can be transferred to uninfected adults during mating (electronic supplementary material, figure S1) [52].

### (b) Butterfly and parasite sources

Adult monarchs were eastern North American migrants collected under USDA APHIS permit number P526P-18-03371 between September and November 2018 near Athens, GA and St Marks, FL, USA. They were kept in overwintering conditions until they were mated in the spring to produce the F1 generation. These F1 offspring were reared in the laboratory using cuttings of greenhouse-grown milkweed (*Asclepias* spp.). Newly emerged F1 uninfected adults were selected to produce three non-inbred F2 lineages used in this experiment. The parasites used were two clonal isolates, E3 and E10, derived from infected wild monarchs in eastern North America and previously characterized in earlier experiments, where they exhibited genetic variation [53]. Parasite strains had been propagated in the laboratory for several generations before this experiment began. We used both isolates in the experimental design to incorporate parasite genetic diversity and to test for strain-specific thermal responses.

### (c) Experimental design

Our experiment followed a fully factorial design across five temperatures, three host lineages and three infection treatments. Ten monarchs per host lineage were inoculated with each of OE isolate E3 and isolate E10, and another ten per host lineage were uninoculated controls, with 90 monarchs in each of the five constant temperature treatments (total *N* = 450 monarchs). First instar larvae were placed in their assigned Percival incubator set at 18, 22, 26, 30 or 34°C with 16 h : 8 h light : dark cycles (incubators used fluorescent lights). Given that past studies have shown signs of declining monarch and parasite fitness at higher temperatures [41,45], we wanted to explore a range of plausible summertime temperatures and responses at the higher end of the monarch thermal range. An iButton (IButtonLink, Whitewater, WI, USA, https://www.ibuttonlink.com/collections/ibuttons) was placed in each incubator to record temperature every 10 min (electronic supplementary material, figure S2). Relative humidity was tracked with a humidity monitor (Fisher Scientific, Waltham, MA, USA), and a pint of water was placed in each of the three hottest incubators to maintain relative humidity above 50%.

To inoculate monarchs, second instar larvae were fed 0.5 cm^2^ pieces of swamp milkweed (*Asclepias incarnata*) onto which 10 parasite spores were manually deposited. Uninoculated control monarchs were fed 0.5 cm^2^ pieces of milkweed with no spores. To ensure parasite spores did not lose viability before being ingested, larvae consumed their inoculum inside individual Petri dishes at room temperature (approx. 21°C). Most caterpillars consumed their inoculum in 6–12 h, and in all cases no longer than 24 h. After inoculation, larvae were returned to their assigned temperature treatments, reared individually in 0.94 l containers and fed fresh cuttings of greenhouse-grown swamp milkweed ad libitum.

### (d) Development, survival and size measures

For each monarch, we measured the time (in days) to pupation and eclosion to assess how temperature and infection influence host development time. We also noted the proportion of monarchs in each treatment group that survived to the pupal and adult stages. After eclosion, adults were placed in 9 cm^2^ glassine envelopes and held at 12°C to measure adult longevity as the difference (in days) between adult emergence and death. Longevity measured at this constant, cooler temperature is a proxy for accumulated energy reserves and survival under reduced metabolism, reflecting the starvation resistance of monarchs reared at different temperatures. The holding temperature of 12°C mimics the cool conditions at the overwintering sites of migratory monarchs, where energy reserves are vital for survival through winter [54]. This post-eclosion metric has been used in prior studies of OE virulence and effects of food limitation [53,55–57].

For monarchs that survived to eclosion, we recorded wing deformity as a binary variable, based on whether one or both wings were crumpled, indicating impaired flight. For adults with intact wings, we used Adobe Photoshop to obtain forewing morphology metrics [58]. Both forewings were scanned at 300 dpi, and we used the right forewing for analysis unless it had notable damage. An image analysis plug-in (FoveaPro 4.0, www.reindeergraphics.com) was used to measure total forewing area (mm^2^). Because roughly half of a monarch's body weight is composed of wing mass, greater wing area can indicate more abundant larval resources [59] and predict adult monarch body size [60].

### (e) Innate immune measures

Each monarch was assigned to one of three haemolymph-sampling groups: bled as larva, bled as pupa or not bled. For fifth instar larvae, we used dissecting scissors to clip a tubercle, collecting 15 µl haemolymph samples. For pupae, we used a 25-gauge needle to prick the dorsal posterior sinus and collect 10 µl haemolymph samples.

For haemocyte counts, haemolymph samples from larvae (3 µl) and pupae (2 µl) were immediately diluted 1 : 10 with sterile Pringle's saline solution (1×, in 1 l of double-distilled (dD) H_2_O: 9.0 g NaCl, 0.2 g KCl, 0.2 g CaCl_2_, 4.0 g dextrose) and loaded into Kova Glasstic haemocytometer slides. We performed haemocyte counts at 400× magnification on two replicate chambers and calculated mean haemocytes per microlitre.

For phenoloxidase (PO) activity assays, an additional 15 µl (larvae) or 7 µl (pupae) of haemolymph was collected per individual (if available), mixed 1 : 1 with ice-cold Pringle's saline, loaded into a microcentrifuge tube, and held at −80°C for 14–20 weeks until we ran the assays. We loaded 10 µl of each sample into 96-well plates with 190 µl of assay buffer (in dD H_2_O: 50 mM Na_2_PO_4_ monobasic monohydrate adjusted to pH 6.5, 2 mM dopamine, and heat-killed *Micrococcus luteus* elicitor at 3% total volume). We measured absorbance at 490 nm every 24 s at 30°C for 338 measures (total run time: 135 min) using a BioTek microplate reader. We recorded the final absorbance value as a proxy for melanization rate because this metric is strongly correlated with slope of the kinetic curve at linear phase [56]. PO assays were run twice for monarchs with additional available haemolymph, and we used the higher final absorbance value of the two for analysis.

### (f) Parasite infection measures

To estimate within-host replication, we examined pupal haemolymph samples at 400× magnification (see above for preparation). For samples with visible OE merozoites (an internal stage of the parasite), we multiplied the average number of merozoites per chamber (based on two replicate chambers) by 100 to estimate number of merozoites per microlitre of haemolymph.

To determine adult infection status, we followed prior methods by pressing 1 cm^2^ pieces of transparent Scotch tape against monarch abdomens and then onto a white index card [30]. We examined the tape samples under 100× magnification, counting spores on the tape. Butterflies were assigned to parasite load classes according to the following scale: 0, no spores; 1, one spore; 2, 2–20 spores; 3, 21–100 spores; 4, 101–1000 spores; and 5, >1000 spores. Prior work indicates that monarchs that score a 1, 2 or 3 likely picked up spores as an adult, while scores of 4 or 5 result from infection at the larval stage [36,52,55]. To include only the monarchs that were infected as larvae, we classified heavily infected adults (score 4 or 5) as parasitized.

Infection probability was measured as the proportion of parasitized adults for each host–parasite–temperature treatment group. To measure infection intensity, we used methods outlined in de Roode *et al*. [40]. After heavily infected adults died, their bodies (following wing removal) were placed into scintillation vials with 5 ml of water. We vortexed the vials for 5 min to dislodge spores and then removed the monarch bodies. Immediately before loading into haemocytometer slides, samples were vortexed for another 30 s and 10 μl aliquots of the mixture were loaded into a Kova Glasstic haemocytometer slide. Mean spore count per 0.1 µl (based on five replicate slide chambers) was multiplied by 5 × 10^4^ to estimate the number of spores per monarch (hereafter, spore load). Spore load is strongly positively correlated with within-host parasite replication [40] and spore transmission [55].

### (g) Statistical analysis

We tested for effects of temperature on monarch fitness, parasite infection and monarch immune defence using generalized additive models (GAMs; method = REML) to capture the nonlinear responses of parasite and host traits to temperature [61,62]. Electronic supplementary material, table S1 describes the structure and error distribution of each GAM. Model residuals were checked for normality and homogeneity of variance, and we confirmed that sufficient basis dimensions were set using the ‘gam.check’ function. As we had just five temperature treatments, we set the number of basis functions in the temperature smooth to five (*k* = 5) but decreased it to four in models that only had responses between 18 and 30°C (merozoite concentration, spore load). *P*-values for both parametric and smoothed terms are reported with a significance level of *α* = 0.05. *R*^2^-adjusted (*R*^2^-adj) is reported as a measure of GAM fit. Models were implemented using the *mgcv* and *stats* packages in the R statistical environment v. 4.0.5 [63,64].

Using only the subset of control (uninoculated, uninfected) monarchs, we employed GAMs to model monarch performance across temperature. Specifically, we tested the responses of development time to adult emergence (log_10_-transformed for normality), survival probability (1 or 0), wing area, probability of wing deformity, and adult starvation resistance (longevity at 12°C). All GAMs included temperature and lineage as smoothed predictors. Monarch lineage was included as a random effect by setting the smooth term's basis spline (s(Lineage, Temperature, bs = ‘re’)), allowing model slopes to vary by lineage. As sex can only be determined at the adult stage, we included sex as a main effect for traits measured after adult emergence. Preliminary analysis indicated that models of wing area should also include bleed treatment as a main effect (electronic supplementary material, figure S3).

To assess whether fitness costs of OE infection varied across temperature treatments, we ran the same GAMs of monarch fitness measures (described above) using data from both uninfected and infected individuals. The uninfected group incorporated both uninoculated monarchs and inoculated monarchs that did not become infected. We included infection status as a main effect along with the interaction between infection and temperature. For survival probability, we used inoculation status since infection status cannot be determined for monarchs that died before the adult stage. To assess whether there were strain-specific effects across temperature, we also ran a subset of models on infected monarch performance, including OE strain and the interaction between strain and temperature. As we did not find evidence that OE strain interacted with temperature, we present the models with infection status in the main text and include details on strain effects in the supplementary material.

To analyse measures of infection, we used data on inoculated monarchs to assess merozoite concentration in pupal haemolymph (log_10_-transformed), infection success (0 or 1) in monarchs inoculated with OE, and the spore load (log_10_-transformed) of infected monarchs. All models included temperature as a smoothed predictor, sex and OE strain as main effects, and lineage as a smoothed random effect. Since host resources can impact parasite replication, the GAM for merozoite concentration also included a smoothed predictor for mass. We used the monarchs sampled for haemolymph to explore responses of immune defence, focusing on haemocyte concentration (log_10_-transformed) and PO activity. Separate models for monarchs sampled as larvae or pupae included temperature and mass as smoothed predictors, lineage as a smoothed random effect, and inoculation status as a main effect. Since the immunity response variables are not specific to OE, we did not include OE strain as a separate main effect. To analyse the impacts of temperature, inoculation, mass and lineage on the proportion of granulocytes, plasmatocytes, oenocytoids or spheroids in larval haemolymph, we used a set of generalized linear models (GLMs; quasibinomial distribution; link = logit) with Bonferroni correction (*α* = 0.0125) to account for multiple comparisons.

To assess effects of temperature on monarch tolerance to infection, we ran linear models of adult starvation resistance (square-root-transformed) as a function of spore load (square-root-transformed), using both infected and uninfected monarchs (as in [65,66]). We included infection status, sex, lineage, area and temperature as main effects in addition to the interaction between spore load and temperature. Finally, we used a series of GLMs to test for trade-offs between immune measures (haemocyte concentration and PO activity) and monarch fitness traits or infection outcomes [67,68].

## 3. Results

**Figure 2.**
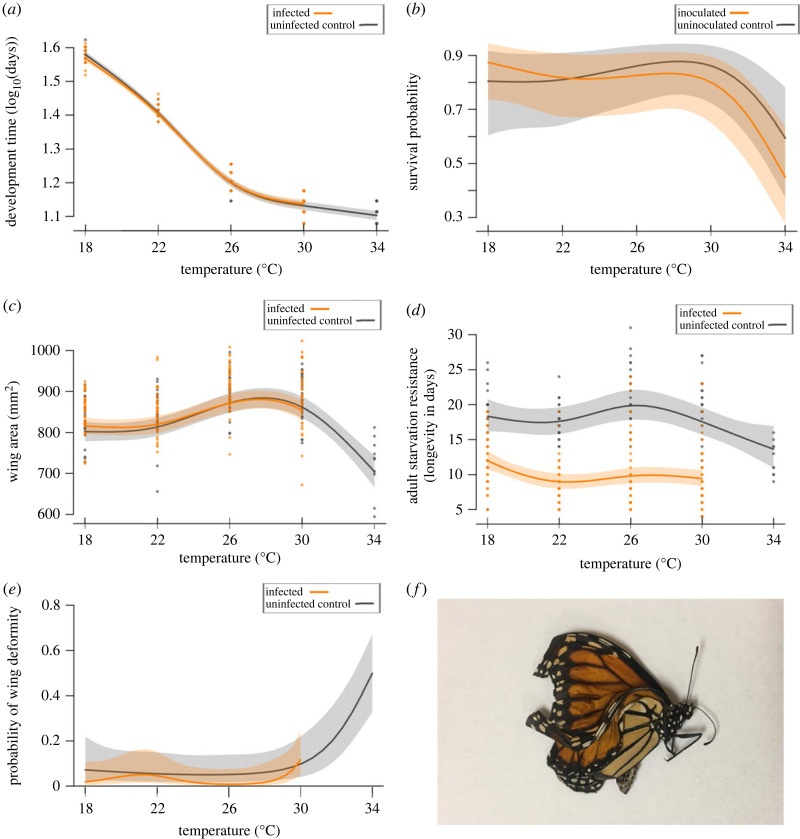
Thermal responses of uninoculated control (grey) and infected or inoculated (orange) monarch performance. We measured (*a*) development time to adult emergence, (*b*) survival probability to the adult stage, (*c*) forewing area, (*d*) adult starvation resistance at 12°C, and (*e*) the probability of wing deformity from 18 to 34°C. The generalized additive model response curves (solid lines), 95% confidence intervals (shaded regions) and raw data points are depicted. (*f*) A deformed, uninfected monarch from the 34°C treatment. Curves depict summed effects; those shown here have sex set as female.

**Figure 3.**
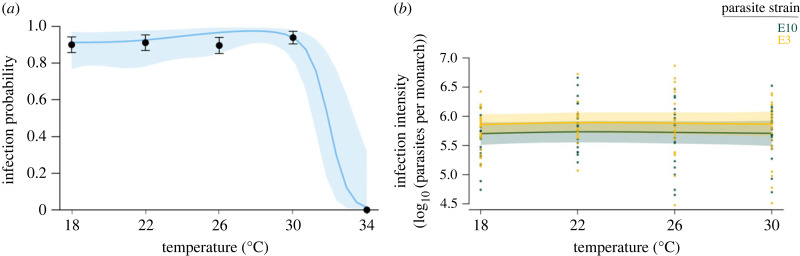
Thermal responses of infection probability and intensity. (*a*) The proportion of inoculated monarchs that emerge infected at the adult stage from 18–34°C, showing a dramatic decrease in infection at the hottest temperature. Black points for mean and standard error of infection probability at each temperature. (*b*) For infected monarchs, parasite load remains consistent from 18 to 30°C, with strain E3 tending to cause more intense infections. In both panels, generalized additive model response curves (solid line) and 95% confidence intervals (shaded region) are shown. Curves depict summed effects with other predictors set as: female; parasite strain E3 in (*a*).

Overall, 78.2% (*n* = 147, s.e. = 3.4%) of the control monarchs and 73.7% (*n* = 300, s.e. = 2.5%) of the inoculated monarchs survived to adulthood (figure 2). Many fewer monarchs survived in the hottest temperature treatment, 47.2% (*n* = 89, s.e. = 5.3%), relative to the four lower temperatures, 82.1% (*n* = 358, s.e. = 2.1%). None of the control monarchs had signs of infection. Of the monarchs inoculated with OE and surviving to the adult stage, 82.2% (*n* = 221, s.e. = 2.6%) became infected, with no infected adults from the 34°C treatment, and over 90% of inoculated monarchs emerging with infection in the other temperature treatments (figure 3).

### (a) Monarch thermal performance with and without infection

Temperature affected each measure of monarch performance. Infected and uninfected monarchs performed very similarly across temperature treatments, with the single major effect of infection observed in adult starvation resistance (infected monarchs had substantially reduced longevity; figure 2).

Development time varied significantly with sex and temperature. Males took slightly longer to develop from hatching to eclosion (*T* = 2.10, d.f. = 1, *p* = 0.04) (electronic supplementary material, table S2). For uninfected control monarchs, development time decreased nonlinearly with temperature, with smaller differences in time to eclosion in the warmer (26–34°C) temperature treatments (*F* = 1500, effective degrees of freedom (ed.f.) = 3.95, *p* < 0.001) (*R*^2^-adj = 0.987) (electronic supplementary material, table S2). Infected monarch development time tracked that of uninfected monarchs very closely (figure 2*a*; electronic supplementary material, tables S2 and S3).

For uninoculated monarchs, the probability of surviving to adulthood decreased at the highest temperature, and one of the monarch lineages had an especially sharp drop in survival at 34°C (*χ*^2^ = 10.3, ed.f. = 2.56, *p* = 0.02) (*R*^2^-adj = 0.225) (electronic supplementary material, table S2). OE inoculation did not cause substantial costs to survival probability relative to the strong effect of temperature (figure 2*b*; electronic supplementary material, tables S2 and S3).

Variation in adult size as measured by wing area was temperature-dependent, but also depended on monarch sex, bleed stage and lineage. Wing area of uninfected monarchs peaked at the intermediate treatments, and then fell sharply at 34°C (*F* = 13.9, ed.f. = 3.70, *p* < 0.001) (*R*^2^-adj = 0.433) (figure 2*c*; electronic supplementary material, table S2). Males had larger wing areas than females (*T* = 2.38, d.f. = 1, *p* = 0.02) and monarchs that were not bled were larger than those bled at the larval or pupal stage (*T* = 2.16, d.f. = 2, *p* < 0.05) (electronic supplementary material, table S2). Wing area did not depend on spore load, infection status, or the interaction between infection and temperature (figure 2*c*; electronic supplementary material, tables S3 and S4).

Uninfected monarch starvation resistance (measured as adult longevity at 12°C) decreased sharply in monarchs reared at 34°C (*χ*^2^ = 31.0, ed.f. = 3.13, *p* < 0.001) (*R*^2^-adj = 0.206) (figure 2*d*; electronic supplementary material, table S2). Starvation resistance of infected monarchs was consistently lower than control individuals from 18 to 30°C (*z* = −11.0, d.f. = 1, *p* < 0.001), but the trend of longevity across temperature did not differ significantly between infected and uninfected monarchs (figure 2*d*; electronic supplementary material, table S3). Notably, although all monarchs at 34°C were uninfected, they had adult lifepans similar to those of infected monarchs at lower temperatures, surviving only 12 days on average (figure 2*d*). Within infected monarchs, those infected with OE strain E3 had lower starvation resistance than monarchs infected with E10 (*z* = −2.78, d.f. = 1, *p* = 0.005), and this pattern was consistent across temperature (electronic supplementary material, figure S4). Notably, we did not find evidence for an interaction between OE strain and temperature for any of the monarch performance metrics (electronic supplementary material, table S5).

The probability of wing deformity in uninfected control monarchs was low across most temperatures but increased markedly at 34°C (*χ*^2^ = 15.7, ed.f. = 2.41, *p* < 0.01) (*R*^2^-adj = 0.175) (figure 2*e*,*f*; electronic supplementary material, table S2). Infection status did not affect the probability of wing deformity, but within infected monarchs, higher spore loads predicted a greater risk of wing deformity (*χ*^2^ = 10.2, ed.f. = 2.33, *p* = 0.017) (electronic supplementary material, tables S3 and S4).

### (b) Infection outcomes by treatment

Infection probability was consistently high at the four lower temperatures and decreased sharply at the hottest temperature, with no successful OE infections in the surviving adults at 34°C (*χ*^2^ = 15.5, ed.f. = 3.56, *p* < 0.01) (*R*^2^-adj = 0.578) (figure 3*a*; electronic supplementary material, table S6). Among the infected monarchs, infection intensity did not depend on temperature and our model did not explain much of the variation in spore load (*R*^2^-adj = 0.073) (figure 3*b*). Males tended to have lower spore loads than females (*T* = −2.42, d.f. = 1, *p* = 0.02), and monarchs infected with strain E3 tended to have higher spore loads (*T* = 2.06, d.f. = 1, *p* < 0.05). The random effect of lineage was a significant smooth term in the model of infection intensity, indicating that host genetics contribute to the severity of OE infection (*F* = 2.52, ed.f. = 1.44, *p* = 0.03) (electronic supplementary material, table S6). Within pupal haemolymph samples, we saw a trend of decreasing merozoite concentration in pupae with increasing temperature (including zero merozoites found at 34°C), although we acknowledge low sample sizes for this measure (electronic supplementary material, figure S5).

### (c) Immunity by treatment

**Figure 4.**
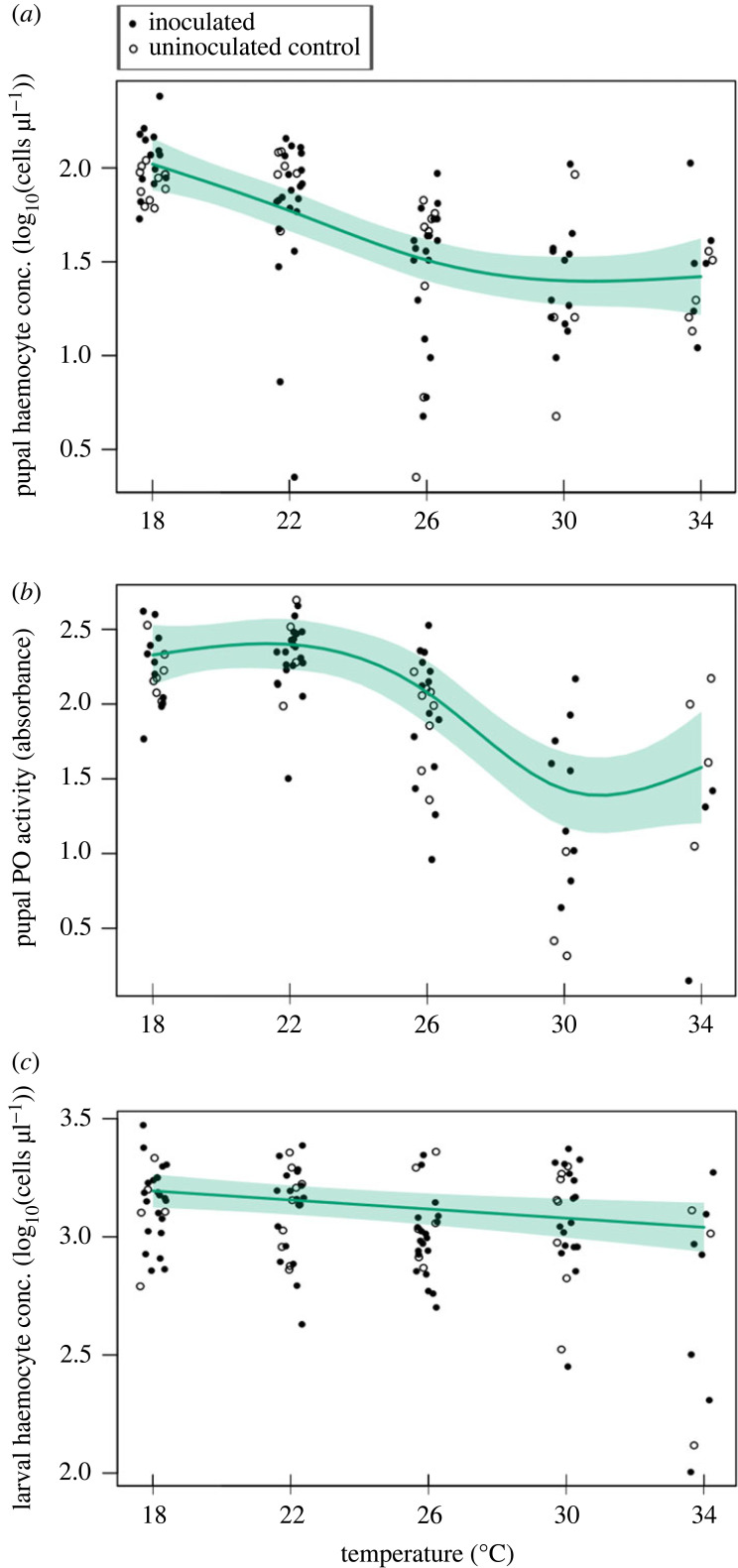
Thermal responses of monarch immune measures from 18 to 34°C. We present (*a*) pupal haemocyte concentration, (*b*) pupal phenoloxidase (PO) activity, and (*c*) larval haemocyte concentration. The generalized additive model response curves (solid lines), 95% confidence intervals (shaded regions), and raw data (open points for control and filled points for inoculated) are shown. Curves depict summed effects with other predictors set to: inoculated and the median mass (approx. 1.3 g).

Haemocyte concentration at the pupal stage decreased with increasing temperature (*F* = 16.9, ed.f. = 2.49, *p* < 0.001) and did not depend on inoculation status (figure 4*a*). Mass and lineage did not impact haemocyte concentration in the pupal stage (*R*^2^-adj = 0.329). Pupal PO activity decreased with increasing temperature (*F* = 19.5, ed.f. = 3.56, *p* < 0.001) and did not depend on inoculation status or mass (*R*^2^-adj = 0.537) (figure 4*b*; electronic supplementary material, table S7).

Larval haemocyte concentrations decreased with warmer temperatures (*F* = 8.49, ed.f. = 1.00, *p* < 0.01) (figure 4*c*). Larval haemocyte concentration increased with mass at the time of bleeding up to a threshold (*F* = 13.8, ed.f. = 2.50, *p* < 0.001) (*R*^2^-adj = 0.396), and inoculation status did not impact haemocyte concentration. Larval PO activity did not depend on temperature but increased with larval mass (*F* = 15.5, ed.f. = 2.02, *p* < 0.001) (*R*^2^-adj = 0.301) (electronic supplementary material, table S7).

Among immune measures we assessed, the random effect of lineage was a significant smooth term in the models of larval haemocyte concentration and pupal PO activity, indicating that host genetics can play a role in monarch immune capacity at various stages (electronic supplementary material, table S7). Temperature, inoculation status, and mass did not significantly explain differences in the proportions of granulocytes, plasmatocytes, oenocytoids or spheroids among larvae (electronic supplementary material, table S8).

### (d) Tolerance across temperature

As shown in prior studies [69], adult monarch starvation resistance (longevity) decreased as infection intensity (spore load) increased (*t* = −2.14, d.f. = 1, *p* = 0.033) (electronic supplementary material, table S9). The steepness of that negative relationship indicates the degree to which infection decreased monarch fitness relative to controls, representing how tolerant monarchs are to infection. Importantly, there were no major differences in the relationship across temperature treatments, as the interaction term between spore load and temperature was not significant (*t* = −0.23, d.f. = 1, *p* = 0.820) (electronic supplementary material, figure S6 and table S9).

### (e) Trade-offs between immunity and fitness

Starvation resistance (adult longevity), development time, wing area and survival probability did not depend on measures of larval or pupal immunity or their interactions with temperature. In this experiment, spore load and infection probability were not predicted by measures of larval or pupal immune defence at any of the experimental temperatures (electronic supplementary material, tables S10–S13).

## 4. Discussion

Quantifying the responses of ectothermic hosts and their parasites to changing environmental temperatures is crucial for predicting the spread and impacts of parasites in a warming world [5]. Findings here showed that sustained heat above the host's thermal optimum reduced monarch fitness—and, more strikingly, that parasites failed to infect any monarchs in the hottest (34°C) treatment. Monarch and parasite fitness generally remained high across a range of temperatures spanning 18 to 30°C, and both monarch performance and parasite infection dropped off steeply at 34°C. This study adds to a growing body of evidence that parasitism in ectotherms might decline under warming in areas that experience extreme and sustained heat [24–26,70], with implications for parasitism in both migratory and non-migratory monarchs.

### (a) High heat reduces parasite infection: examples and mechanisms

Steep drops in parasite fitness in inoculated hosts at high temperatures have been reported for several other ectotherm–parasite systems. Primary examples come from fungal entomopathogens, whose hosts can limit infections through behavioural fever (e.g. [30]). Our study, in conjunction with past work, suggests that this pattern also occurs in some apicomplexan parasites of insects. Studies of *Plasmodium* spp*.* in mosquitoes and *Gregarina cubensis* in cockroaches suggest that prolonged exposure to high heat can hinder parasite development [71–73]. Early work by MacDougall [74] found that, after 6 days at 37.5°C, mealworm hosts cleared gregarine parasite infections; this clearance was not due to faster encapsulation (i.e. improved host immunity) at higher temperatures, but rather resulted from heat killing the parasite's trophozoite stage. Together, these studies suggest that some ectothermic hosts could escape infection with warming, motivating additional studies of the within-host mechanisms underlying reduced parasite performance.

We found that inoculated monarchs reared at 34°C emerged as uninfected adults, with no signs of OE spores. This lack of infection did not result from higher monarch immune defence or faster monarch development at the hottest temperature. For some insects, immune metrics like haemocyte concentration and PO activity increase at higher temperatures and play a role in parasite inhibition [29,30]. In other systems, including another butterfly species, higher temperatures have been found to decrease immunity [31,32]. Here, measures of monarch immunity were either consistent across temperature or decreased in the hottest treatments. Thus, it is unlikely that monarch immune defence caused the failure of OE to infect at 34°C. One limitation of this experiment is that we did not investigate all possible stage-specific impacts of temperature across the monarch life cycle. Early instar larvae are most susceptible to OE [39,75], so temperature-dependent immunity at earlier stages might better predict infection outcomes. Notably for monarchs, although the lower measures of general immunity at higher temperatures did not impact OE outcomes, monarchs might be more vulnerable to other infectious agents.

As monarchs showed similarly fast development at 30 and 34°C, it seems unlikely that monarch development outpaced OE at higher temperatures. In fact, parasite spore loads were very similar across the full 18–30°C range, despite faster host development at warmer temperatures. It could be that the constant 34°C temperature treatment exceeded the thermal maximum of parasite replication and survival. A prior study reported decreased longevity and viability of parasite spores when stored at warmer temperatures (up to 32°C), with the optimal temperature for survival of the dormant parasite transmission stage between 4 and 12°C [45]. Pinpointing the specific mechanism that caused infections to fail at 34°C will require future experiments that quantify early instar immunity, parasite developmental progression across temperature, and monarch–parasite responses to a range of extreme-heat exposure times.

### (b) Consequences of host responses for parasite fitness under extreme heat

Beyond direct effects of heat in reducing parasite replication and survival, results here show that high temperatures will further reduce parasite fitness through negative impacts on host fitness. In particular, extreme heat reduced monarch immature survival probability, adult longevity and wing size, and increased the likelihood of monarch wing deformity. Past work demonstrated that these host fitness traits are crucially important for parasite lifetime fitness [53]. First, if monarchs do not survive to eclosion, OE cannot be transmitted to a new host. In addition, any reduction in monarch adult longevity, mating probability or flight performance also means fewer opportunities for transmission (including vertical transmission, environmental transmission via spore deposition on host plant leaves, and adult–adult transfer of spores) [76]. In the hottest treatment (34°C), monarchs were 35% less likely to survive to the adult stage, experienced a 42% reduction in adult longevity, had 17% smaller wings, and were 53% more likely to have deformed wings. As demonstrated by prior experiments and field studies [40,53,55,77], these lower host fitness metrics will decrease the probability of parasite transmission under extreme heat.

### (c) Stable parasite virulence across temperature

The degree to which OE caused harm to monarchs was consistent across the 18–30°C temperature range. Here, we found that the clearest negative impact of infection on monarchs was to reduce adult starvation resistance (longevity), consistent with past work [40,53,56]. Below 34°C, infection lowered starvation resistance by approximately 40%, irrespective of temperature. Importantly, spore load and the spore load–monarch longevity relationship did not vary across the four lower temperature treatments. By contrast, past work showed that environmental conditions experienced by the parasite's external stages over longer time spans (e.g. several weeks to many months) affect both spore load and the degree of harm that OE parasites cause to monarchs [45]. Moreover, different OE genotypes are known to vary dramatically in both virulence and within-host replication [55], and both parasite and host genotypes interact to determine infection probability [69]. Our findings of similar virulence metrics across a range of temperatures indicate that any future changes in parasite virulence are unlikely to be driven by thermal responses of the within-host dynamics of infection.

### (d) Range-wide implications of extreme heat

Past observational studies showed high prevalence of OE infections in the warmer (more southerly) parts of the monarch breeding range [36,78,79]. Our study suggests that nighttime warming and heatwaves that exceed the thermal limits of OE could shift the location of transmission hotspots and the seasonal timing of infection. OE prevalence has increased threefold over the past two decades [80], in part owing to warmer winters supporting resident monarch populations along the Gulf Coast, which typically have high prevalence owing to year-round breeding [79]. However, recent record-breaking high temperatures indicate that continued warming in the southeast USA could lead to reductions in summer infection prevalence through decreased host and parasite survival, as well as through indirect temperature effects like reduced availability and increased toxicity of milkweeds [81,82]. By contrast, average warming at the northern edge of the migratory monarch breeding range is less likely to limit parasite infection, and these regions could experience higher peak prevalence through a longer breeding season. Alternatively, some studies predict that greater climate variability and temperature anomalies at higher latitudes could cause heat stress for ectotherms [83]. In northern parts of the monarch breeding range, temperature variation could result in more frequent extreme heat events that surpass the thermal limit of OE within-host replication, leading to temporary local parasite extinction.

Spatiotemporal overlap between migratory and resident monarchs occurs during migration, allowing parasite transfer between residents and migrants during spring breeding [84]. If warming expands the area of resident breeding in the southern USA, shifts the timing of peak infection prevalence earlier, or selects for more heat-tolerant OE strains, migratory monarchs could experience higher infection risk during and following their northward spring movements [84]. Additional work is urgently needed to understand how multiple axes of host and pathogen responses to warming will interact to affect host–pathogen dynamics and evolution, both in the southern USA (with year-round monarch breeding) and in the monarchs' more northerly seasonal breeding range.

### (e) Conclusions and future directions

In contrast to assumptions of the Thermal Mismatch Hypothesis [2,23], where parasites are expected to have a wider thermal range and thus outperform hosts away from the host's thermal optimum, our findings demonstrate that at temperatures above the host's optimum, within-host processes can limit parasite performance such that warming reduces infection. Both monarchs and OE spores are known to survive in habitats where midday temperatures surpass 34°C [44,84,85], likely because they experience relief from the heat at night, and through behavioural thermoregulation where caterpillars seek shade, thus altering the internal temperature environment experienced by OE [86]. Future studies should therefore use fluctuating temperatures, simulated heatwaves, or shorter pulses of extreme temperatures to assess impacts on infection outcomes [15,44,87–89]. Additionally, experiments should assess whether parasite strains from different locations or infecting different host populations (e.g. migratory versus resident monarchs) demonstrate distinct thermal performances, as might result from differential selection pressures on parasites. Finally, studies should address other temperature-sensitive variables that could influence host immunity or parasite fitness, such as food plant quality and chemistry [57,81,90,91]. These approaches could inform transmission models that account for multiple underlying effects of temperature, to better predict the responses of monarch–parasite interactions to warming across a broad and heterogeneous geographical range.

## Data Availability

The data are available in the Dryad Digital Repository: https://doi.org/10.5061/dryad.tht76hf4r [92]. Code and data used in this study are also available in a GitHub repository: https://github.com/IRagonese/MonarchOE_Temperature_Infection2019.git. The model outputs are provided in the electronic supplementary material [93].
